# Supplementary material for: Inhibition of oxidative stress in cholinergic projection neurons fully rescues aging-associated olfactory circuit degeneration in Drosophila
Source: eLife. 2018 Jan 18;7:e32018. doi: 10.7554/eLife.32018 (PMC5790380; doi:10.7554/eLife.32018)
Supplement: Supplementary file 1. [file elife-32018-supp1.docx]

|  | **GO.ID** | **Term** | **Annotated** | **Significant** | **Expected** | **classicFisher** |
| --- | --- | --- | --- | --- | --- | --- |
| *7* | GO:0006508 | proteolysis | 398 | 119 | 74.87 | 1.3e−08 |
| *1* | GO:0006952 | defense response | 235 | 83 | 44.21 | 5.7e−10 |
| *19* | GO:0002376 | immune system process | 279 | 80 | 52.48 | 2.3e−05 |
| *3* | GO:0009607 | response to biotic stimulus | 226 | 78 | 42.51 | 6.6e−09 |
| *4* | GO:0043207 | response to external biotic stimulus | 226 | 78 | 42.51 | 6.6e−09 |
| *5* | GO:0051707 | response to other organism | 226 | 78 | 42.51 | 6.6e−09 |
| *8* | GO:0006955 | immune response | 181 | 64 | 34.05 | 5.8e−08 |
| *14* | GO:0098542 | defense response to other organism | 168 | 58 | 31.60 | 6.3e−07 |
| *6* | GO:0009617 | response to bacterium | 145 | 56 | 27.28 | 1.2e−08 |
| *10* | GO:0042742 | defense response to bacterium | 124 | 47 | 23.33 | 3.5e−07 |
| *27* | GO:0042335 | cuticle development | 119 | 37 | 22.39 | 0.00076 |
| *29* | GO:0045087 | innate immune response | 112 | 35 | 21.07 | 0.00094 |
| *15* | GO:0006959 | humoral immune response | 80 | 34 | 15.05 | 7.1e−07 |
| *16* | GO:0006022 | aminoglycan metabolic process | 66 | 29 | 12.42 | 2.1e−06 |
| *26* | GO:0050776 | regulation of immune response | 84 | 29 | 15.80 | 0.00042 |
| *23* | GO:0019730 | antimicrobial humoral response | 67 | 26 | 12.60 | 9.6e−05 |
| *30* | GO:0031347 | regulation of defense response | 76 | 26 | 14.30 | 0.00097 |
| *18* | GO:0006030 | chitin metabolic process | 54 | 24 | 10.16 | 1.2e−05 |
| *21* | GO:1901071 | glucosamine−containing compound metaboli... | 57 | 24 | 10.72 | 3.8e−05 |
| *22* | GO:0006040 | amino sugar metabolic process | 59 | 24 | 11.10 | 7.3e−05 |
| *9* | GO:0050830 | defense response to Gram−positive bacter... | 40 | 22 | 7.52 | 3.0e−07 |
| *20* | GO:0019748 | secondary metabolic process | 50 | 22 | 9.41 | 3.5e−05 |
| *17* | GO:1901136 | carbohydrate derivative catabolic proces... | 39 | 20 | 7.34 | 4.5e−06 |
| *25* | GO:0009620 | response to fungus | 45 | 19 | 8.47 | 0.00023 |
| *2* | GO:0006026 | aminoglycan catabolic process | 23 | 18 | 4.33 | 9.6e−10 |
| *11* | GO:0006032 | chitin catabolic process | 17 | 13 | 3.20 | 3.8e−07 |
| *12* | GO:0046348 | amino sugar catabolic process | 17 | 13 | 3.20 | 3.8e−07 |
| *13* | GO:1901072 | glucosamine−containing compound cataboli... | 17 | 13 | 3.20 | 3.8e−07 |
| *24* | GO:0044550 | secondary metabolite biosynthetic proces... | 16 | 10 | 3.01 | 0.00014 |
| *28* | GO:0005984 | disaccharide metabolic process | 8 | 6 | 1.50 | 0.00086 |
