# Supplementary material for: Inhibition of oxidative stress in cholinergic projection neurons fully rescues aging-associated olfactory circuit degeneration in Drosophila
Source: eLife. 2018 Jan 18;7:e32018. doi: 10.7554/eLife.32018 (PMC5790380; doi:10.7554/eLife.32018)
Supplement: Supplementary file 2. [file elife-32018-supp2.docx]

|  | **GO.ID** | **Term** | **Annotated** | **Significant** | **Expected** | **classicFisher** |
| --- | --- | --- | --- | --- | --- | --- |
| *27* | GO:0044281 | small molecule metabolic process | 436 | 125 | 63.37 | 3.8e−16 |
| *28* | GO:1901135 | carbohydrate derivative metabolic proces... | 291 | 92 | 42.29 | 9.8e−15 |
| *26* | GO:0019637 | organophosphate metabolic process | 233 | 91 | 33.86 | 1.5e−21 |
| *22* | GO:0055086 | nucleobase−containing small molecule met... | 181 | 82 | 26.31 | 1.6e−24 |
| *29* | GO:0055114 | oxidation−reduction process | 244 | 81 | 35.46 | 2.3e−14 |
| *24* | GO:0009117 | nucleotide metabolic process | 164 | 77 | 23.84 | 2.8e−24 |
| *25* | GO:0006753 | nucleoside phosphate metabolic process | 165 | 77 | 23.98 | 4.6e−24 |
| *16* | GO:0072521 | purine−containing compound metabolic pro... | 151 | 76 | 21.95 | 2.0e−26 |
| *17* | GO:0009150 | purine ribonucleotide metabolic process | 141 | 72 | 20.49 | 1.5e−25 |
| *18* | GO:0006163 | purine nucleotide metabolic process | 142 | 72 | 20.64 | 2.6e−25 |
| *19* | GO:0009259 | ribonucleotide metabolic process | 143 | 72 | 20.78 | 4.5e−25 |
| *20* | GO:0019693 | ribose phosphate metabolic process | 144 | 72 | 20.93 | 7.8e−25 |
| *1* | GO:0046034 | ATP metabolic process | 101 | 68 | 14.68 | < 1e−30 |
| *2* | GO:0009126 | purine nucleoside monophosphate metaboli... | 104 | 68 | 15.12 | < 1e−30 |
| *3* | GO:0009167 | purine ribonucleoside monophosphate meta... | 104 | 68 | 15.12 | < 1e−30 |
| *4* | GO:0009144 | purine nucleoside triphosphate metabolic... | 105 | 68 | 15.26 | < 1e−30 |
| *5* | GO:0009205 | purine ribonucleoside triphosphate metab... | 105 | 68 | 15.26 | < 1e−30 |
| *6* | GO:0009161 | ribonucleoside monophosphate metabolic p... | 106 | 68 | 15.41 | < 1e−30 |
| *7* | GO:0009199 | ribonucleoside triphosphate metabolic pr... | 106 | 68 | 15.41 | < 1e−30 |
| *8* | GO:0009123 | nucleoside monophosphate metabolic proce... | 108 | 68 | 15.70 | < 1e−30 |
| *9* | GO:0009141 | nucleoside triphosphate metabolic proces... | 108 | 68 | 15.70 | < 1e−30 |
| *21* | GO:0006091 | generation of precursor metabolites and ... | 124 | 66 | 18.02 | 8.0e−25 |
| *12* | GO:0045333 | cellular respiration | 95 | 60 | 13.81 | 3.7e−28 |
| *23* | GO:0015980 | energy derivation by oxidation of organi... | 106 | 60 | 15.41 | 1.6e−24 |
| *10* | GO:0022900 | electron transport chain | 71 | 52 | 10.32 | 2.4e−29 |
| *11* | GO:0022904 | respiratory electron transport chain | 69 | 51 | 10.03 | 4.0e−29 |
| *14* | GO:0006119 | oxidative phosphorylation | 65 | 48 | 9.45 | 2.0e−27 |
| *15* | GO:0042773 | ATP synthesis coupled electron transport | 63 | 47 | 9.16 | 3.4e−27 |
| *13* | GO:0042775 | mitochondrial ATP synthesis coupled elec... | 60 | 46 | 8.72 | 1.6e−27 |
| *30* | GO:0032543 | mitochondrial translation | 90 | 39 | 13.08 | 2.2e−11 |
